# Identification of a small optimal subset of CpG sites as bio-markers from high-throughput DNA methylation profiles

**DOI:** 10.1186/1471-2105-9-457

**Published:** 2008-10-27

**Authors:** Hailong Meng, Edward L Murrelle, Guoya Li

**Affiliations:** 1 Scientific Division, ClearPoint Resources Inc., Richmond, VA 23219, USA; 2 Life Sciences Research, Altria Client Services, Richmond, VA 23219, USA

## Abstract

**Background:**

DNA methylation patterns have been shown to significantly correlate with different tissue types and disease states. High-throughput methylation arrays enable large-scale DNA methylation analysis to identify informative DNA methylation biomarkers. The identification of disease-specific methylation signatures is of fundamental and practical interest for risk assessment, diagnosis, and prognosis of diseases.

**Results:**

Using published high-throughput DNA methylation data, a two-stage feature selection method was developed to select a small optimal subset of DNA methylation features to precisely classify two sample groups. With this approach, a small number of CpG sites were highly sensitive and specific in distinguishing lung cancer tissue samples from normal lung tissue samples.

**Conclusion:**

This study shows that it is feasible to identify DNA methylation biomarkers from high-throughput DNA methylation profiles and that a small number of signature CpG sites can suffice to classify two groups of samples. The computational method we developed in the study is efficient to identify signature CpG sites from disease samples with complex methylation patterns.

## Background

DNA methylation, which occurs when a methyl (CH_3_) group is added at the carbon 5 position of the cytosine ring of a CpG dinucleotide, is one of the epigenetic events that can affect gene expression without changing genomic sequence [1]. For example, hypermethylation of CpG sites in the promoter region was implicated as playing a role in the inactivation of tumor suppressor genes [2,3]. DNA methylation patterns have been shown to significantly correlate with clinical phenotypes [4-6]. DNA methylation signatures are excellent biomarker candidates because: 1) distinct DNA methylation profiles correspond to different tissue types and disease states, and each type or subtype of tumor has its own DNA methylation signature [5,7]; 2) DNA methylation patterns change at early stages of disease progression, allowing earlier detection of diseases [8]; 3) DNA methylation can be detected with high sensitivity [9]; 4) DNA methylation biomarkers could be detected from peripheral bio-fluid [10,11], such as blood, when it is not possible to obtain disease-tissue samples from patients. The identification of disease-specific methylation signatures is therefore of fundamental and practical interest for risk assessment, diagnosis, and prognosis of diseases.

High-throughput methylation arrays are now available to determine DNA methylation levels of thousands of CpG sites, simultaneously [4,5,12-14]. This technology enables large-scale DNA methylation analysis to identify informative DNA methylation biomarkers. For example, experiments using high-throughput methylation arrays have demonstrated that each of colon, breast, lung, and prostate cancer cell lines has its own methylation signature [5]. It has also been shown that DNA methylation profiles could clearly distinguish human embryonic stem cells from cancer cells, adult stem cells, lymphoblastoid cells, and normal cells [4]. Additionally, Bibikova et al. [5] identified 55 CpG sites as the DNA methylation signature to distinguish normal lung tissue samples from lung cancer tissue samples.

Although the profiles from high-throughput methylation arrays contain a large number of CpG sites, many of them are irrelevant or redundant and provide little discriminatory information to classify samples. For clinical diagnosis, significant savings in cost can be achieved by measuring and verifying methylation levels of only a small number of CpG sites. Recent studies showed that a small discriminative set of features was sufficient to better classify samples in high-throughput gene expression analysis [15,16].

The Support Vector Machine (SVM) is a state-of-the-art classification method (classifier or predictor) [17] that has been widely used in microarray data analysis [18-21]. Although the SVM was designed to deal with datasets in high-dimensional space [17], it has continued to suffer from the "curse of dimensionality", that is, learning from a small number of samples in a high-dimensional feature space [21]. Including redundant and non-informative features in the analysis may cause the influence of discriminatory features to be lost in the noise, thus degrading the accuracy of the classifier. A large feature set may achieve low training error, but the ability to generalize the new dataset will decrease, resulting in data overfitting [22].

Classification methods can be improved by feature selection, a process designed to select a small, optimal subset of features from the original redundant feature set. In general, feature selection methods fall into two categories: filter methods and wrapper methods [23]. Filter methods select features independent of the classification method. One typical filter method is individual feature ranking, which is straightforward, computationally efficient, and widely used for gene selection in gene expression data analysis [24-26]. However, this method has several limitations. First, feature redundancy is common in the selected feature set and many features carry essentially the same discriminatory information. In addition, this strategy does not detect dependencies among features and lacks the ability to determine which combination of features achieves the best classification since individual feature ranking evaluates each feature independently. In contrast to filter methods, wrapper methods work with classifiers to determine feature selection based on the predictive accuracy of the classifiers [18,21]. Although wrapper methods generally outperform filter methods, they are typically computationally intensive [23] and may become intractable in practice for large feature sets. SVM_RFE (Recursive Feature Elimination) is a typical wrapper method that has displayed excellent prediction ability in microarray data analysis [18,21]. Genetic algorithms (GAs) have been employed as feature selection methods in high-throughput biological data analysis [27-29], but are very time-consuming.

In this study, we investigated whether a small number of signature CpG sites are sufficient to predict phenotypic classes of two sample groups. A biomarker discovery algorithm was developed. This algorithm, here referred to as FW_SVM, uses a two-stage feature selection method by combining a Filter method and a Wrapper method and employs SVM as the classifier.

## Methods

### Datasets

We used three published datasets generated by the Illumina GoldenGate^® ^assay for DNA methylation (Illumina, San Diego, CA), where the reported β value indicates the methylation level of each CpG site [4,5]. The first dataset included the DNA methylation profiles from 19 male and 25 female cell lines. The second dataset contained the DNA methylation profiles of 37 human embryonic stem cell (hES) and 24 cancer cell lines. The third dataset contained 23 lung adenocarcinoma and 23 normal lung tissue samples, 11 each from Philipps University of Marburg (Germany) and 12 each from the Pennsylvania State University College of Medicine Tumor Bank.

The data in each dataset were split into training and testing sets. The training set was used for feature selection and classifier training, and the testing set was used to evaluate algorithm performance.

### Feature selection methods

#### FW_SVM

**Figure 1:**
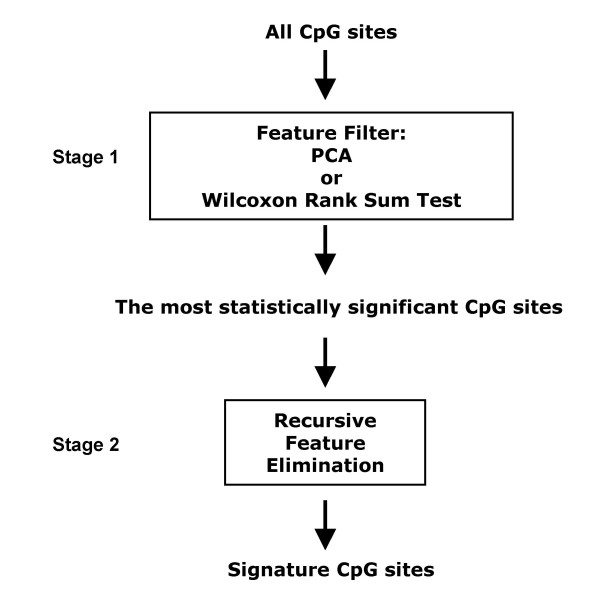
**Two-stage feature selection method of FW_SVM.** In the first stage, Principal Component Analysis (PCA) and Wilcoxon rank sum test were tested separately as the feature filter. The RFE algorithm was used to select the signature feature set in the second stage.

We developed a two-stage feature selection method (Figure 1) for FW_SVM, and it takes advantage of both filter methods and wrapper methods. In the first stage of feature selection, the filter method removes most of the statistically unimportant features and selects a list of the most important CpG sites as signature candidates. In the second stage, SVM_RFE (the algorithm of SVM_RFE is described below) is used to remove redundant features and select the smallest feature set achieving the best classification as signature CpG sites.

Two filter methods for stage 1, namely Principal Component Analysis (PCA) and Wilcoxon rank sum test, were tested separately in this study. PCA [30] is a multivariate method that has been widely used for visualization of high-dimensional data, including high-throughput biological data [31], in low-dimensional space. PCA is seldom used for feature selection since each principal component is a linear combination of all original features and does not isolate or prioritize features. However, since the first several principal components typically capture most of the variability in the data, features that have big projections on those principal components account for the major source of data variance. Accordingly, those features are likely good candidates as signature features for classification purposes. In the first filter method, when PCA was applied at the first feature selection stage, CpG sites with an absolute loading value greater than 0.1 for the first 10 principal components were selected as signature feature candidates taken by SVM_RFE at the second stage. In the second filter method, we adopted the Wilcoxon rank sum test. In comparison to the PCA approach of selecting features with large variances across the entire dataset, the individual feature ranking targeted directly the classification goal and selected a list of the most differentially methylated CpG sites as promising feature candidates. The CpG sites from Wilcoxon rank sum test were sorted by their p-values in ascending order. The top 50 most differentially methylated CpG sites were selected as signature feature candidates with a restriction that the differences of methylation level (*β value*) means between two groups were greater than 0.15.

The feature selection method of FW_SVM was compared with two popular feature selection methods: individual feature ranking and SVM_RFE.

#### Individual feature ranking

Individual feature ranking selects features according to their individual relevance. Its implementation is simple and requires minimal run time. In this experiment, all CpG sites were ranked in ascending order based on their p-value from the Wilcoxon rank sum test. The Wilcoxon rank sum test can be applied to data from any distribution and is robust to outliers. An additional filter was applied to remove CpG sites whose mean differences of methylation level (*β value*) between two groups were less than 0.15. The top-ranked 1, 2, 3, 5 or 10 of the most differentially methylated CpG sites were selected as signature CpG sites.

#### SVM_RFE

Recursive Feature Elimination (RFE) is a backward feature selection method designed to find the best combination of features for classification. Less important features, in terms of the predictive accuracy of SVM, are successively eliminated, allowing for the selection of only the best subset of features. The RFE algorithm is outlined below:

##### Initialization

*F *= [1, 2, ..., *n*] is the subset of remaining features.

*R *= [] is the subset of ranked features.

##### Feature selection

1. For *k *= 1, 2, ..., *n *remove the *k*th feature and evaluate the cross-validation error on the reduced feature set using the training dataset.

2. Remove the feature with maximum cross-validation error and include it to the top of *R*.

3. Repeat 1 and 2 for remaining features in *F*, until *R *contains all ranked features.

SVM_RFE is an application of RFE using SVM as the classifier in the feature selection process [21]. In this study, leave-one-out cross-validation was employed to evaluate the classification performance of each feature set. Each sample was excluded from the training set, one at a time, and then classified based on the SVM trained from the remaining samples. This procedure was repeated, in turn, for all samples, and the cross-validation error was defined as the sum of misclassifications. In the process, cross-validation error vs. the size of the feature set was recorded, and the smallest subset of features with the least cross-validation error was chosen as the final methylation signature.

### Classification method

We selected SVM as the classification method to evaluate signature features selected from different feature selection approaches. Note that both SVM_RFE and FW_SVM also took SVM as classifiers in their feature selection process.

SVM is a supervised machine learning technique to solve classification problems [17]. It maps the data into a higher dimensional space and constructs an optimal hyperplane to maximize the separation margin between two classes. In this study, we adopted Least Square SVM (LS-SVM), a modified version of SVM that benefits from its computational simplicity and efficiency by solving a set of linear equations instead of quadratic programming [32]. In a training dataset of n samples, {*x*_*i*_, *y*_*i*_}, *i *= 1, 2, ..., *n*, where *x*_*i *_∈ *R*^*d *^is a sample point with *d *features, and *y*_*i *_∈ {-1, +1} indicates the class of a sample. The class label of a new sample is obtained by a decision function:

(1) $$f(x)=sign\left(\sum_{i=1}^{n}\alpha_{i}y_{i}K(x,x_{i})+b\right)$$

where parameters *α*_*i *_and *b *are optimized in the training procedure such that the number of misclassifications on the training set is minimized. *K*(*x*_*i*_, *x*) is a kernel function.

The LS_SVMlab toolbox  was used in the implementation of LS_SVM [33], and the RBF kernel function with default parameters (*γ *= 10 and *σ*^2 ^= 0.2) was adopted.

### Performance testing and evaluation

Each of the three DNA methylation datasets generated by Illumina high-throughput DNA methylation arrays [4,5] was split into training and testing sets. The training set contained approximately 2/3 of the samples and the testing set included the remaining 1/3. The feature selection methods were performed on training datasets. To validate the features selected by each method, raw SVMs learned from methylation profiles of the signature CpG sites in the training set, and the trained SVMs were used to predict the phenotypic classes of the samples in the testing set.

In order to minimize bias introduced by data partitioning and to accurately assess performance of the feature selection methods, each dataset was randomly partitioned into training and testing sets multiple times. For individual feature ranking and FW_SVM, the sensitivity, specificity, accuracy, number of signature features, and running time reported for each dataset represent the average across 100 independent runs. SVM_RFE was very time-consuming with each run requiring several days to complete. Therefore, its reported performance results are from only 5 random partitions of training and testing datasets.

Sensitivity, specificity and accuracy were used to assess the performance of classification:

(2) $$Sensitivity=\frac{TP}{TP+FN}$$

(3) $$Specificity=\frac{TN}{TN+FP}$$

(4) $$Accuracy=\frac{TP+TN}{TP+FN+TN+FP}$$

where *TP*, *FP*, *TN *and *FN *represent true positives, false positives, true negatives and false negatives, respectively.

### Pathway analysis

Pathway Studio™ [34] with database Resnet 5.0 was used to build gene interaction pathways from a list of genes whose upstream CpG sites were differentially methylated.

All computational methods (except Pathway Studio) in this study were implemented in MATLAB (The MathWorks, Inc., Natick, MA) and run on a PC with a 3.8 GHz CPU and 3.0 GB RAM.

## Results and discussion

### Comparison and discussion of feature selection methods

**Figure 2:**
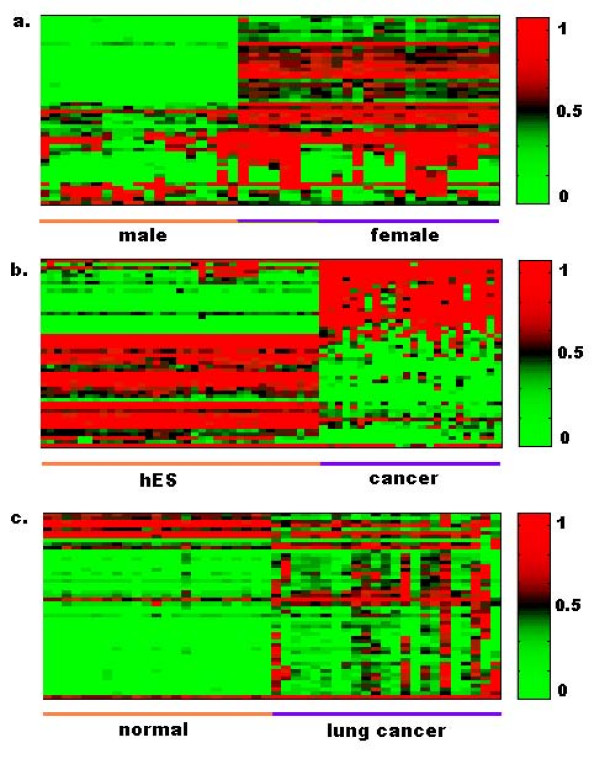
**Heat maps of the 50 most differentially methylated CpG sites in three datasets.** The green color indicates low methylation level and red indicates high methylation level as shown in the colormap on the right side. (a) Male and female cell line samples. (b) hES and cancer cell line samples. (c) Normal and lung cancer tissue samples.

Figure 2 displays heat maps of the methylation status for the 50 most differentially methylated CpG sites in the three datasets, simply obtained from Wilcoxon rank sum tests. Many CpG sites are methylated at different levels among male and female cell lines (Figure 2a), reflecting differential epigenetic regulation patterns by gender. Of the top 25 most differentially methylated CpG sites, 21 are on the X-chromosome. Many genes display distinct methylation profiles between cancer cell lines and hES cell lines (Figure 2b), although it has been reported that cancer cells share some characteristics with hES cells [35]. Considering only one feature is sufficient to obtain high prediction accuracy for the cancer vs. hES cell line dataset and for the male vs. female cell line dataset (Table 1). The individual feature ranking method works well for the datasets with distinct methylation patterns.

**Table 1:** Performance results of individual feature ranking

Dataset	N*	Sensitivity (%)	Specificity (%)	Accuracy (%)	Run Time (seconds)
Male and female cell lines	1	99.4	100	99.7	0.18
	2	99.8	100	99.9	0.18
	3	100	100	100	0.18
	5	100	100	100	0.18
	10	100	100	100	0.18

Cancer and hES cell lines	1	95.0	99.9	98.0	0.34
	2	96.1	99.9	98.5	0.35
	3	96.6	100	98.7	0.35
	5	99.6	99.8	99.8	0.35
	10	100	99.5	99.7	0.35

Lung cancer and normal tissues	1	73.0	91.9	82.4	0.19
	2	81.1	91.1	86.1	0.20
	3	84.1	90.0	87.1	0.20
	5	88.8	91.6	90.2	0.20
	10	92.1	89.6	90.9	0.20

*N: The average number of selected signature CpG sites.

In contrast, the lung cancer and normal tissue data (Figure 2c) show different results. Perhaps due to the intrinsic complexity of disease mechanisms, the lung cancer tissue samples exhibited highly variable methylation patterns. In the present case, the methylation profile of a single CpG site is not sufficient to achieve accurate separation between normal and lung cancer samples (Table 1). An ideal DNA methylation signature, therefore, would consist of a small subset of CpG sites to provide non-redundant and complementary discriminative information.

SVM_RFE is a backward feature selection method and was designed to find an optimal combination of features by eliminating less-important features successively. However, due to its lengthy run times (Table 2), the computational cost of SVM_RFE likely limits its practical use in high-throughput data analysis.

**Table 2:** Performance results of SVM_RFE and FW_SVM for lung cancer and normal tissue dataset

Method	N*	Sensitivity (%)	Specificity (%)	Accuracy (%)	Run Time
SVM_RFE	3.6	95.0	85.0	89.9	5.1 days
FW_SVM with PCA	2.6	89.1	94.3	91.7	8.7 minutes
FW_SVM with individual feature ranking	2.2	87.4	94.1	90.8	8.4 minutes

*N: The average number of selected signature CpG sites.

**Figure 3:**
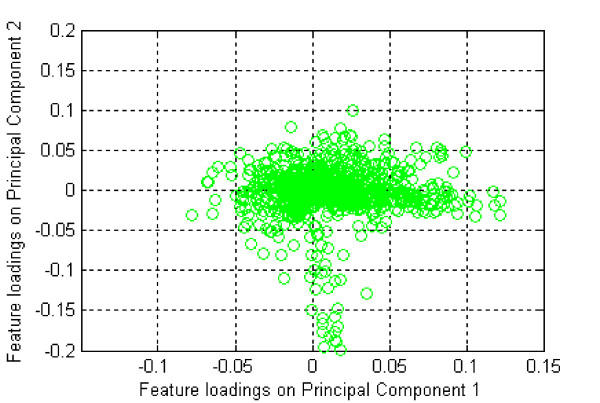
**Loading plot of 1596 CpG sites on the first two principal components for normal and lung cancer tissue dataset in PCA analysis.** Most features cluster around the origin point, indicating that they contribute very little to the first two principal components.

In FW_SVM, a two-stage feature selection method was developed. The irrelevant and noisy information was eliminated by a filter at the first stage, and then SVM_RFE was used to detect the final optimal feature subset from the remaining informative features at the second stage. The first applied filter is PCA. Analysis of the normal and lung cancer tissue dataset using PCA (Figure 3) found that the loadings of most features fell near the origin, suggesting that these features are likely not important nor informative for classification, while features that have big projections on those principal components account for the major source of data variance and are likely good candidates as signature features for classification purposes. We also tested individual feature ranking (Wilcoxon rank sum test in the study) as the first-stage feature selection method in the implementation of FW_SVM, in which we selected the 50 most differentially methylated CpG sites as feature candidates based on an estimated balance between feature coverage and computational cost. FW_SVM provides the flexibility to select either a smaller or a larger candidate feature set for distinguishing two sample groups according to specific datasets or a user's preferences. In comparison with individual feature ranking and SVM_RFE, as shown in Table 2, both versions of FW_SVM achieved similar or better performance with more compact feature size and a 2000-fold shorter run time than SVM_RFE. By identifying and utilizing the complementary discriminative information in the signature feature set, FW_SVM obtained similar or better predictive accuracy with only approximately two non-redundant CpG sites as did individual feature ranking with 10 CpG sites.

Without other available DNA methylation datasets, FW_SVM was tested on a benchmark microarray gene expression dataset [25]. The profiles of two genes identified by FW_SVM can classify Acute Myeloid Leukemia (AML) and Acute Lymphoblastic Leukemia (ALL) sample groups with the average accuracy of 98.8% (data not shown).

### An application of FW_SVM: signature CpG sites identification to classify lung cancer and normal tissue samples

The DNA methylation profiles in this study displayed excellent biomarker characteristics. Accurate discrimination between two sample groups was achieved on the basis of only a few CpG sites. In order to compare our results with signature CpG sites obtained by Bibikova et al. [5], we applied FW_SVM (the individual feature ranking version in this experiment) to identify signature CpG sites for normal and lung cancer tissue samples. We used 11 normal samples and 11 adenocarcinoma samples from the Philipps University of Marburg (Germany) as our training set and 12 normal samples and 12 adenocarcinoma samples from the Pennsylvania State University College of Medicine Tumor Bank as the testing set. From the training set, FW_SVM selected two CpG sites, TNF-1371 and TWIST1-524, as signature features. Based on those two signature CpG sites, the predictor correctly classified all of the normal and lung cancer tissue samples in the testing set and achieved better sensitivity and specificity than the 55 CpG site markers identified by Bibikova et al. [5].

To further verify the reliability of these two signature CpG sites, we mixed the samples from these two datasets together and randomly split them 100 times into a training set (containing 2/3 of the samples) and a testing set (containing 1/3 of the samples). Raw SVMs were trained on the profiles of these two CpG sites in the training sets, and trained SVMs were used to predict the phenotype of samples in the testing sets. The average sensitivity achieved was 96%, and the average specificity was 100%.

**Figure 4:**
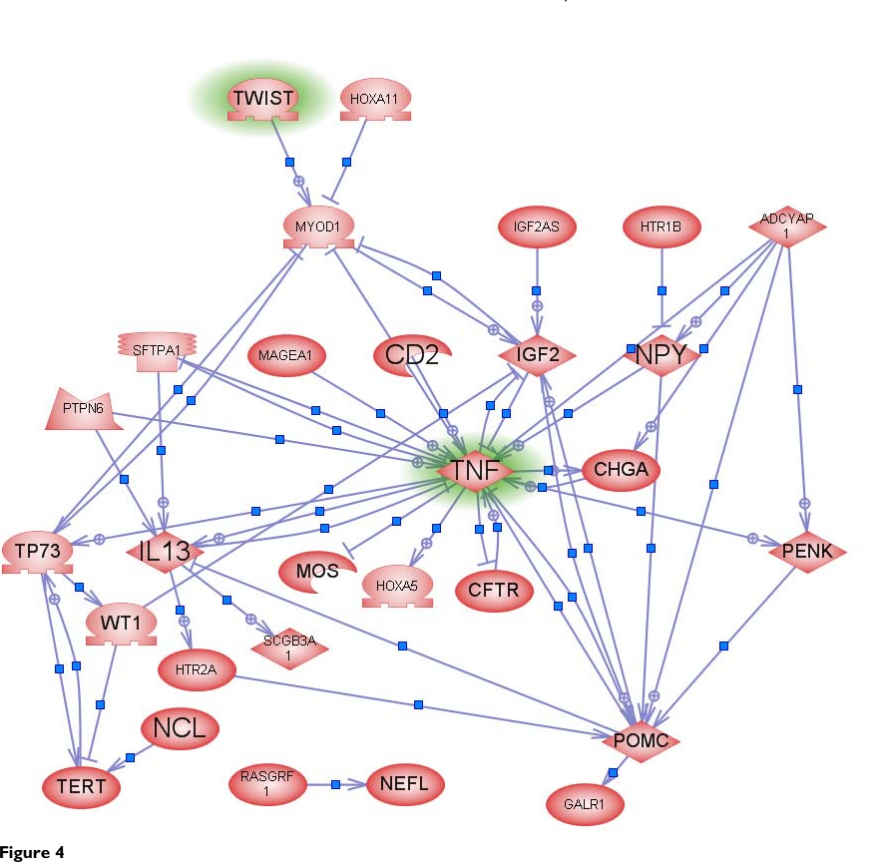
Pathway analysis of genes that contain the 100 most differentially methylated CpG sites between normal and lung cancer tissue samples (p-value < 0.005). Genes that do not connect to other genes are not shown in this figure.

We also investigated the biological pathway in which the genes containing those two signature CpG sites are involved. Given that many factors influence gene expression, DNA methylation changes do not necessarily translate to changes of gene expression [36,37]. However, it remains very likely that genes with differentially methylated CpG sites are involved in the development of lung cancer. Pathway Studio™ software was used to analyze the genes with the 100 most differentially methylated CpG sites between normal and lung cancer tissue samples (p-value < 0.005 and mean difference > 0.15). This analysis detected direct expression interactions among many of those genes. As shown in Figure 4, TNF, a multifunctional proinflammatory cytokine that belongs to the tumor necrosis factor super family [38], is one of the hub genes in this network. Its expression level, controlled by methylation regulation, may have a critical influence on other genes. This may explain why the methylation status of a CpG site in the promoter region of TNF is critical for classifying normal and lung cancer samples. TWIST, a basic helix-loop-helix transcription factor implicated in cell lineage determination and differentiation [39], is found upstream of TNF in this network and its role in cancer has been studied broadly [39-42].

In this study, we identified the smallest subset of CpG sites required for precise classification of lung cancer and normal tissue samples, with every signature CpG site containing necessary, non-redundant and mutual information in the context of others. All the signature CpG sites identified are important biologically, but it is not necessary to include all important CpG sites for classification purposes.

While these two signature CpG sites (TNF-1371 and TWIST1-524) are promising leads for potential diagnostic purposes, they were detected from a relatively small dataset of 46 samples. Accordingly, the reliability of the TNF and TWIST CpG sites as biomarkers for lung cancer requires further validation in larger datasets and through targeted biological experiments.

### Patterns vs. profile distances

**Figure 5:**
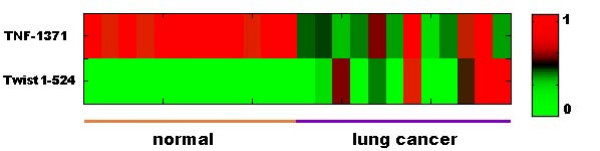
**DNA methylation profiles of two signature CpG sites (TNF-1371 and TWIST1-524) from 12 lung cancer and 12 normal tissue samples in the testing dataset.** The green color indicates low methylation level and red indicates high methylation level as shown on the right of the colormap.

Figure 5 displays the DNA methylation profiles of TNF-1371 and TWIST1-524 from 12 lung cancer and 12 normal tissue samples in the testing dataset. The profiles of normal tissue samples are generally uniform and consistent, while the profiles of cancer samples are highly variable. This likely reflects the biological complexity of cancer. To classify samples from different groups, a classification method should have the ability to recognize all the patterns belonging to each group. Hierarchical clustering (HC) is generally used for class prediction in gene expression analysis. However, HC is neither an appropriate nor effective classification method. First, HC is considered an unsupervised technique, since no sample class information is utilized in the clustering. Thus, the output from HC is simply a clustering tree, and no class information about new samples is provided. Secondly, HC splits samples into groups on the basis of similarity measured by distances of profiles. However, classification is a pattern-identification problem, and the distances between profiles do not exactly reflect the real patterns leading to the classification. As shown in Figure 5, lung cancer samples have diverse patterns, and it is neither possible to cluster them nor to distinguish them from normal tissue samples purely based on distances among methylation profiles, regardless of the distance measurement (correlation or Euclidean distance) and linkage method (single, complete, or average) used. FW_SVM, on the other hand, employs SVM, a supervised machine learning technique, as the classification method. Instead of using profile distance, SVM is able to learn and draw patterns for different classes from labeled training samples. By capturing the essentials of the pattern recognition, SVM provides a generally accurate class prediction, as shown in this study.

## Conclusion

This study shows that it is feasible to identify DNA methylation biomarkers from high-throughput DNA methylation profiles and that a small number of signature CpG sites can suffice to classify two groups of samples. Signature CpG sites can easily be detected from datasets with clear methylation patterns, such as male and female datasets, using traditional feature selection methods like individual feature ranking. However, the traditional feature selection methods were not efficient to identify signature CpG sites from disease samples with complex DNA methylation patterns, such as the lung cancer tissue examined in this study. We investigated two filter methods for SVM_RFE in the study and built up FW_SVM, a predictor with an efficient feature selection method. FW_SVM was able to detect a small, optimal subset of CpG sites with non-redundant and complementary discriminative information and achieved high predictive accuracy to classify disease samples with complex DNA methylation patterns. Since each CpG site represents a feature, and the methylation level of each CpG site simply corresponds to the value of the feature, the FW_SVM algorithm, in principle, could be extended to analyze other post-genomic datasets, such as high-throughput gene expression, microRNA expression, single nucleotide polymorphisms, and proteomic data, individually or even across platforms, to identify combinatorial signature features. Therefore, FW_SVM represents a highly flexible tool that can be adopted in classification situations in which appropriate high-throughput data are available to potentially aid in diagnosis and gain fundamental insight into disease processes.

## Availability and requirements

**Project name**: FW_SVM

**Project home page**: None. Matlab scripts for FW_SVM were submitted to BMC Bioinformatics as additional file 1.

**Operating system**: platform independent

**Programming language**: Matlab

**Other requirements**: Work together with LS-SVMlab toolbox that can be downloaded from: 

**License**: None

**Any restrictions to use by non-academics**: None

## Authors' contributions

HM developed and implemented the algorithm under the supervision of GL and ELM. The initial manuscript draft was written by HM, and refined by GL and ELM.

## Supplementary Material

Additional file 1 **Matlab scripts for FW_SVM.**  FW_SVM is a biomarker discovery algorithm and it can identify a small optimal subset of CpG sites from high-throughput DNA methylation profiles to distinguish two sample groups. FW_SVM combined Filter method and Wrapper method as a novel two-stage feature selection method and employed SVM as the classifier. Click here for file
